# The Brassinosteroid Signaling Pathway—New Key Players and Interconnections with Other Signaling Networks Crucial for Plant Development and Stress Tolerance

**DOI:** 10.3390/ijms14058740

**Published:** 2013-04-24

**Authors:** Damian Gruszka

**Affiliations:** Department of Genetics, Faculty of Biology and Environment Protection, University of Silesia, Jagiellonska 28, Katowice 40-032, Poland; E-Mail: damian.gruszka@us.edu.pl; Tel.: +48-32-2009-482; Fax: +48-32-2009-396

**Keywords:** arabidopsis, brassinosteroids, cross-talk, hormones, pathogen-resistance, signaling, stress tolerance

## Abstract

Brassinosteroids (BRs) are a class of steroid hormones regulating a wide range of physiological processes during the plant life cycle from seed development to the modulation of flowering and senescence. The last decades, and recent years in particular, have witnessed a significant advance in the elucidation of the molecular mechanisms of BR signaling from perception by the transmembrane receptor complex to the regulation of transcription factors influencing expression of the target genes. Application of the new approaches shed light on the molecular functions of the key players regulating the BR signaling cascade and allowed identification of new factors. Recent studies clearly indicated that some of the components of BR signaling pathway act as multifunctional proteins involved in other signaling networks regulating diverse physiological processes, such as photomorphogenesis, cell death control, stomatal development, flowering, plant immunity to pathogens and metabolic responses to stress conditions, including salinity. Regulation of some of these processes is mediated through a crosstalk between BR signalosome and the signaling cascades of other hormones, including auxin, abscisic acid, ethylene and salicylic acid. Unravelling the complicated mechanisms of BR signaling and its interconnections with other molecular networks may be of great importance for future practical applications in agriculture.

## 1. Introduction

Brassinosteroids (BRs) are plant-specific polyhydroxylated steroid hormones displaying high activity in stimulation of plant growth and development and regulation of a broad spectrum of physiological responses to biotic and abiotic stress conditions. The first identified representative of BRs was steroidal lactone—brassinolide, extracted from pollen grains of *Brassica napus* and displaying high level of biological activity [1,2]. BRs have been isolated in a broad range of species representing various evolutionary groups [3,4]. Extensive genetic and biochemical research, conducted over the last two decades, mainly in *Arabidopsis thaliana*, led to the elucidation of BR biosynthesis as well as identification and functional analysis of the signaling components [5]. It has been reported that BR biosynthesis potentially occurs in every plant organ and its level is developmentally regulated, and contrary to other plant hormones, BRs do not undergo long-distance transport [6,7]. BRs have been found at relatively high concentrations in pollen grains and immature seeds, whereas mature organs contain much lower concentration of these hormones. Defects in BR metabolism often result in plant height reduction to various extents. Semi-dwarfism of crop plants has long been recognized as indispensable trait in breeding of many monocot crops and is still of great importance for agriculture [5,8].

BRs regulate a broad range of physiological processes, such as: seed development and germination, cell division and elongation, what results in highly stimulating impact on plant growth, differentiation of tracheary elements, polarization of cell membrane, proton pumping to apoplast and into a vacuole by stimulation of transmembrane ATPases, as well as increasing the efficiency of photosynthesis by elevating the level of CO_2_ assimilation and Rubisco activity. BRs stimulate the expression of alfa- and beta-tubulin genes and affect reorientation of cortical microtubules, which influence arrangement of cellulose microfibrylls. Leaf senescence proved to be stimulated by this group of hormones as well. When exogenously applied at high concentrations, BRs inhibit root elongation, but promote lateral roots formation. Moreover, BRs regulate the processes of photo- and skotomorphogenesis (etiolation) and are known to have a positive impact on reproductive development and regulation of flowering time [5]. Many of these physiological processes are regulated based on network of interactions with auxins [9–13].

Recently BRs have gained more attention as numerous reports indicated that BRs modulate plant metabolic response to environmental biotic and abiotic stresses, and these physiological processes include: salt and drought stress tolerance, thermotolerance, oxidative stress tolerance, pathogen resistance, as well as herbicide and pesticide tolerance [14]. The question how BRs may impact such a diverse physiological processes remained unanswered. However, an extensive amount of data gathered in genetic, biochemical and physiological assays enabled characterization of BR signal transduction pathway initiated by hormone perception by plasma membrane-associated receptor complex, transduced *via* phosphorylation/dephosphorylation cascade to the regulation of gene expression by a group of transcription factors. It has been reported that some of the components of BR signaling are directly involved in other molecular pathways regulating various physiological processes. Thus, these protein factors may function as cross-talk points interconnecting BR signaling pathway with the molecular mechanisms regulating various aspects of plant physiology. This paper reviews recent advances in elucidating molecular mechanisms of BR signal transduction pathway including introduction of new key players of this relay and presents molecular mechanisms of interconnections of BR signaling pathway with other molecular networks.

## 2. Brassinosteroid Perception by Plasma Membrane-Associated Receptor Complex

Recently by deploying genetics, genomics, proteomics and many other approaches performed mainly in *Arabidopsis thaliana* a model of BRs signal transduction pathway has been established. The process is commenced by the perception of the hormone ligand by the cell membrane-associated receptor complex, which initiates a relay mediated by phosphorylation/dephosphorylation cascade leading to changes in target gene expression [15]. In plants, the major class of receptors encompasses Receptor-like Kinases (RLKs) with about 600 members in Arabidopsis [16,17]. BRs are directly perceived by the transmembrane polypeptide BRI1 (Brassinosteroid-Insensitive1), which belongs to the vast family of Leucine-Rich Repeat Receptor-like Kinases (LLR-RLK) encompassing more than 200 protein kinases in Arabidopsis [16,18–20]. In Arabidopsis BRI1 is an 1196 amino acid-long protein, localized in both the plasma membrane and endosomes, and is ubiquitously present in all organs [21,22]. An average density of BRI1 receptors in the plasma membrane equals to 12 receptors μm^−2^, and differences in BRI1 density in the plasma membranes of different tissues may be of regulatory importance and contribute to various sensitivities to BR [23,24]. Since its first description, more than 30 different alleles of *BRI1* gene have been identified [25]. This receptor kinase is composed of three major parts: extracellular LRR domain, single-pass transmembrane domain and cytoplasmic kinase domain. The N-terminal part of BRI1 protein contains signal peptide followed by leucine zipper motif and pair of cysteines. The last two elements are responsible for homo- and heterodimerization [26]. It has been reported that BRI1 can form ligand-independent homodimers in plasma membranes [27,28], however BRI1 homodimerization was shown to be promoted or stabilized by BR [29]. This part of BRI1 protein is followed by 25 tandem Leucine-Rich Repeats, each of them spanning 24 amino acids, forming secondary structures of α-helises and β-sheets [30]. There is 70-amino acid island located between 21st and 22nd LRRs, being responsible together with the 22nd LRR for binding of BR molecules, forming 94-amino-acid-long steroid-binding motif [31]. The part of the BRI1 protein comprising the LRRs mediates interactions with other polypeptides [32,33] and forms a right-handed superhelix of twisted LRRs with the 70-amino acid domain folding into the interior of the superhelix to form a binding pocket for a single BR molecule per BRI1 monomer [34]. Stoichiometry and specificity of the ligand binding by BRI1 monomer is determined by the restricted size of the ligand-binding pocket and its hydrophobic nature [35]. A high-resolution model of the extracellular part of Arabidopsis BRI1 receptor has been recently determined. Direct binding of the BR molecule form a docking platform for co-receptor, leading to initiation of signaling relay [35,36]. Downstream of the LRR domain, a single-pass transmembrane domain spanning amino acids 792–814 is located, followed by cytoplasmic juxtamembrane domain (JM) covering amino acids 815–882. The protein region from Phe-883 to Phe-1155 comprises the kinase domain (KD). The protein fragment spanning amino acids 1156–1196 is the carboxy-terminal segment of intracellular part [37].

## 3. BR Ligand Binding by BRI1 Receptor Is Accompanied by Numerous Phosphorylation Events, Which Have Regulatory Function

Mass spectrometry analysis allowed identification of numerous phosphorylation sites in cytoplasmic fragment of BRI1, located throughout the juxtamembrane, kinase and C-terminal domains [38,39]. Phosphorylation initiated by BR perception in the activation loop of the kinase domain is essential for stimulation of the kinase activity, whereas phosphorylation within juxtamembrane and C-terminal domains quantitatively enhances BRI1 activity [40]. Apart from autophosphorylation on specific serine and threonine residues in the juxtamembrane region, kinase domain (including activation loop residues Ser-1044 and Thr-1049 being crucial for BRI1 kinase function) and C-terminal domain, it was recently reported that BRI1 autophosphorylates on tyrosine residues as well [39]. Autophosphorylation is hierarchical and proceeds as follows: phosphoserine–phosphothreonine–phosphotyrosine. Therefore, BRI1 was categorized as a dual-specificity LRR-RLK. It was recently reported that juxtamembrane domain of BRI1 is an activator of kinase activity and that kinase autophosphorylation specificity in terms of target residues (Ser, Thr or Tyr) is influenced by residues outside of the kinase domain. Interestingly, autophosphorylation of residues within the juxtamembrane domain precedes the autophosphorylation of majority of residues in the kinase domain, except for the residues located within the activation loop, which are required for kinase catalytic activity and are therefore phosphorylated as the first [17,41]. In addition to autophosphorylation on tyrosine residues, BRI1 may also phosphorylate downstream substrates on tyrosine residues [42–44]. Phosphorylation on Tyr-831 in the juxtamembrane domain of BRI1 enhances BR signaling. On the contrary, several tyrosine residues within the kinase domain of BRI1 (*i.e.*, Tyr-1052, Tyr-1057) stimulate its kinase activity only in de-phosphorylated state [39,41]. It has been recently reported that autophosphorylation of Ser-891 in ATP-binding domain of BRI1 is one of the deactivation mechanisms that inhibit BRI1 activity and repress BR signaling. The rate of autophosphorylation of this residue increases slowly after BR perception [45]. There are two mechanisms that operate to inhibit basal BRI1 activity in the absence of BR: C-terminal domain of BRI1 functions to suppress its kinase activity, and secondly BKI1 protein (described later) binds to BRI1 to prevent its association with other components of BR receptor complex [29,46].

Binding of BR molecule by BRI1 receptor domain encompassing the 70-amino acid island and the downstream LRR induces conformational alterations in the receptor (preformed homodimer or BRI1 homodimerization), causing autophosphorylation within the kinase domain activation loop, what results in stimulation of the kinase activity, what brings about the association and phosphorylation of the second component of receptor complex, what renders it active [35,44]. BR binding triggers conformational changes of the BRI1 extracellular LRR domain to expose dimerization interface for binding the LRR domain of the co-receptor, but also initiates the conformational change of the BRI1 kinase domain to activate its function leading to transphosphorylation. This in turn causes repression of the autoinhibitory action of BRI1 C-terminal domain and release of the BKI1 protein, which allows for receptor complex formation. This is so called ‘double-lock’ mechanism, in which both the extracellular LRR domain and the kinase domain take part, and which is crucial for assembly of the fully active receptor complex and efficient downstream signaling [47,48]. It is suggested that BRI1-Supressor1 (BRS1), which is a serine carboxypeptidase may participate in functioning of the extracellular domain of BRI1 receptor, especially in enzymatic processing of peptide components involved in BR perception [49,50].

## 4. BRI1 Forms Receptor Complex through Interaction with SERK Transmembrane Kinases What Involves Further Phosphorylation Events

It was shown that heterodimerization of BRI with four members of the Somatic Embryogenesis Receptor Kinase (SERK) subfamily of LRR-RLKs is required for full activation of the signaling pathway. Initially activated by ligand binding BRI1 can not activate downstream signaling components without previous transactivation with SERK proteins [51,52]. The members of the SERK gene family have emerged by gene duplication event, which was followed by maintaining of functional redundancy among the paralogues [53]. The SERK proteins, and BRI1-Associated receptor Kinase1/Somatic Embryogenesis Receptor-like Kinase3 (BAK1SERK3) in particular, interact directly with BRI1. Upon binding to BRI1 receptor they become transphosphorylated and activated. The activated SERKs transphosphorylate BRI1 at the juxtamembrane and C-terminal domain to fully trigger BRI1 kinase activity [44]. Transphosphorylation of both polypeptides transactivates them and is stimulated by BR [20,54,55]. The sequential phosphorylation model is now well established. BR binding most likely causes conformational alteration of the receptor, inducing BRI1 autophosphorylation in the activation loop, which activates BRI1 kinase domain. Active BRI1 kinase interacts with BAK1/SERK3 (or other proteins of the SERK family presented below) and phosphorylates the co-receptor in the activation loop, leading to their activation. The SERK co-receptors in turn phosphorylate the juxtamembrane and C-terminal domain of BRI1, which causes its full activation [40,53]. This model suggests that members of the SERK family are required for full activation of the BRI1 receptor, but are not involved in ligand binding [44].

Auto- and transphosphorylations cause exposition of the sites of interaction with other components of the signaling pathway, what enables formation of the receptor complex and initiates the signal transduction [56]. The BRI1 protein contains sixteen autophosphorylation sites, four of them are located within the juxtamembrane domain, ten are situated within the kinase domain and two in C-terminal region. Moreover, there are three transphosphorylation sites in the juxtamembrane domain and two in the C-terminal domain. The BAK1 co-receptor contains two autophosphorylation sites in the kinase domain and three in the C-terminal domain, additionally this protein contains six transphosphorylation residues located within the kinase domain, four out of them are situated within the activation loop [53]. BAK1/SERK3 kinase also possesses amino-acid residues within the kinase domain, like kinase activation loop residue Thr-455, corresponding to BRI1 residue Thr-1049, when phosphorylated stimulate the enzymatic activity of BAK1/SERK3 and are crucial for the function in BR signaling [38,54].

**Figure 1:**
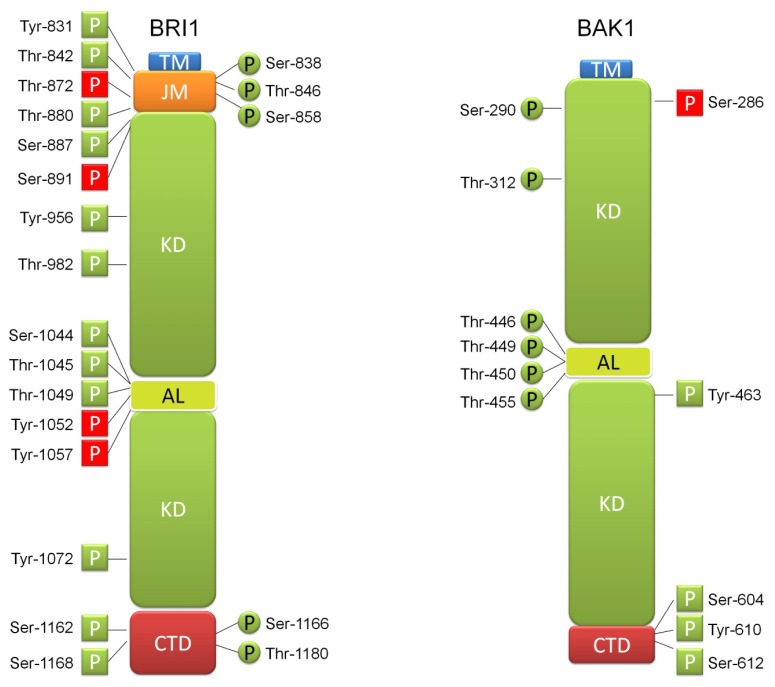
Pattern of auto- and transphosphorylation sites of the BRI1 and BAK1 proteins. Autophosphorylation sites are shown as squares, whereas transphosphrylation sites as solid circles containing the letter ‘P’. Green symbols represent residues, whose phosphorylation has stimulatory effect on the activity of the BRI1 and BAK1 proteins, whereas red symbols denote phosphorylated residues with inhibitory impact on the activity of the BRI1-BAK1 receptor complex. Abbreviations: TM—transmembrane domain, JM—juxtamembrane region, KD—kinase domain, AL—activation loop, CTD—C-terminal domain.

Similarly to BRI1, BAK1/SERK3 also functions as a dual-specificity kinase and phosphorylates on tyrosine residues, what influences a subset of BAK1/SERK3 activities [44,57]. Phosphorylations on tyrosine residues play important role in functional activity of BAK1. Phosphorylation of Tyr-463 within BAK1 kinase domain is essential for its catalytic activity, whereas phosphorylation of Tyr-610 in the carboxyl-terminal domain of BAK1 is also a major site of autophosphorylation, which is BR-induced and indispensable for its kinase activity and for the stimulation of BRI1 through transphosphorylation. Interestingly, Tyr-610 phosphorylation is required for BR signaling and innate immunity, but not for suppression of programmed cell death and flagellin signaling (mediated by FLS2) aimed at inhibition of growth, indicating that pattern of phosphorylation is very important for distinct activities of the BAK1 kinase in various signaling pathways [57]. It is suggested that both Ser/Thr and Tyr phosphorylations are important for the kinase activities of BRI1 and BAK1/SERK3, whereas Tyr phosphorylation is crucial for specific BR responses mediated by various signaling pathways [44]. Apart from positive impact of phosphorylation on the activities of BRI1 and BAK1, it was also reported that phosphorylation on specific residues, like Thr-872 in BRI1 and Ser-286 in BAK1 may negatively regulate their function [38,40]. The auto- and transphosphorylation sites of the BRI1 and BAK1 proteins are shown in Figure 1.

## 5. Mechanisms Regulating the Activity of BRI1-SERKs Receptor Complex

The transmembrane BRI1-BAK1 heterodimer functioning as BR co-receptor is asymmetric—BAK1/SERK3 is shorter than BRI1 because contains only five LRRs and does not include the pair of cysteines and 70-amino acid island, therefore BRI1 protein is the only component of the receptor complex that binds BR ligands [31,32,58]. It has been demonstrated that BRI1-BAK1/SERK3 heterodimerization leads to endocytosis of cell membrane fragments containing these polypeptides, what may constitute the mechanism facilitating the interaction of the receptor complex with cytoplasmic enzymes functioning as components of the signal transduction pathway [22,27,33,58]. Endocytosis may play a significant role in mediating receptor recycling and function [59]. Endocytosis of BR receptor complex is particularly important in the roots, where water may dilute the extracellular BR molecules. In fact, it was observed that plasma membrane-bound BRI1-BAK1 complexes are found in most cells, however endosomal fraction of these polypeptides was detected mainly in root cells [22].

Recently another mechanism of regulation of the BRI1 receptor kinase activity has been reported. Dephosphorylation of BRI1 by protein phosphatase 2A (PP2A), whose distinct function in BR signaling will be described later, causes down-regulation of BRI1 activity and signaling pathway. This process is suggested to be regulated by BRs through stimulation of the Suppressor of bri1 (SBI1) leucine carboxy-methyltransferase, which methylates the PP2A phosphatase facilitating its interaction with BRI1, what results in dephosphorylation of the receptor kinase [60,61].

On the other hand, activity of BAK1 kinase is regulated by interaction with the Membrane Steroid-Binding Protein1 (MSBP1). MSBP1 is capable of binding the BR ligands, however with lower affinity than BRI1, and interacts specifically with the extracellular LRR domain of BAK1 in BR-independent manner. It is suggested that this interaction suppresses BR signaling. MSBP1 enhances BAK1 endocytosis, thus shifts the equilibrium to endosomes. Function of MSBP1 is based on preventing the interactions between BRI1 and BAK1, therefore MSBP1 acts as a negative regulator of the early events during BR signal transduction. Enhanced BAK1 endocytosis triggered by association with MSBP1 causes reduction in BAK1-stimulated BRI1 endocytosis, what results in BR signaling inhibition. It is suggested that BR-hypersensitive phenotype of the *elongated* (*elg*) mutant, which carries a mutation in the extracellular region of BAK1 protein, may be caused by reduced MSBP1-BAK1 interactions, what results in attenuated suppression of BAK1 activity [59].

## 6. SERKs Are Multifaceted Co-Receptors Mediating Various Signaling Pathways

It has also been shown that BAK1/SERK3 kinase interacts with other LRR-RLKs to induce their functions and initiate other signaling pathways that regulate plant defense responses [62–66]. This kinase is also involved in negative regulation of cell-death control [57]. This indicates that BAK1/SERK3 is a multifaceted factor functioning as a co-receptor in several independent pathways by stimulating the signaling relay of various LRR-RLKs, which bind diverse ligands [47,63,67,68]. It is proposed that the previously described ‘double-lock’ mechanism enables versatile BAK1 protein to associate with various LRR-RLKs binding different ligands and form the stable receptor-coreceptor complexes through physical interactions of their kinase domains to ensure efficient transphosphorylation. Moreover, this mechanism enables regulation of this dimerization procedure by both extra- and intracellular cues [48]. BAK1/SERK3 mediates distinct signaling pathways most probably also through specific pattern of its site-specific phosphorylation [57].

Other members of the SERK family including SERK1, SERK2 and SERK4/BKK1 (BAK1-like) also interact directly with BRI1 receptor and redundantly promote BR signaling, however SERK2 plays a minor role in this process [52]. Apart form mediating BR signaling SERK1 and SERK2 regulate anthers development [69,70], whereas SERK4/BKK1 is also implicated in the suppression of cell death [71,72]. Despite this redundancy in SERK family, BAK1 is up to now the only member of this family directly involved in the innate immunity and pathogen response [63,73]. This phenomenon has significant implications for the inter-connections between BR signaling and other molecular relays regulating crucial physiological processes, and will be presented in further section of this review.

## 7. Downstream BR Signaling Components Interacting Directly with Receptor Complex

Several new proteins participating in the assembling of the BR receptor complex have recently been identified. One of these components that regulates the BR signaling is BKI1 (BRI1 Kinase Inhibitor1)—negative regulator, which in the absence of BR is a membrane-bound protein anchored in the plasma membrane by N-terminal lysine-arginine-rich motif, whereas its C-terminal domain binds specifically to the cytoplasmic C-terminal domain of BRI1 receptor [46,74]. It is suggested that the association with BR receptor is mediated through the conserved 20-amino acid C-terminal domain of BKI1. This protein-protein association most likely inactivates BRI1 function through preventing its interactions with BAK1/SERK3 polypeptide and other receptor components, and blocking the signal transduction at the cell membrane level [58,74]. Binding the BR molecule by BRI1 receptor causes dissociation of BKI1 polypeptide and initiates formation of the receptor complex [33,46]. It is mediated by the phosphorylations on Tyr-211, Ser-270 and Ser-274 of BKI1, which are catalyzed by BRI1 [74,75]. However, it has been recently reported that BKI1 plays also a positive role in the BR signaling by binding to a subset of 14-3-3 proteins (function of the 14-3-3 proteins will be discussed later). The C-terminal domains of the phosphorylated, cytosolic BKI1 proteins, released from the plasma membrane upon BR binding, associate with the 14-3-3 proteins and repress their negative functions in BR signaling [76]. It results in accumulation of active transcription factors, regulating expression of BR-responsive genes. Phosphorylation of Ser-270 and Ser-274 is essential for the binding of BKI1 to 14-3-3 proteins [75].

A proteomic analysis led to the identification of other components of the BR receptor complex—BR-Signaling Kinases (BSKs) belonging to the subfamily of the Receptor-like Cytoplasmic Kinases (RLCK-XII) and functioning as positive regulators of BR signaling. The members of BSK family transmit the signal between membrane-bound receptor complex and cytoplasmic regulators of BR signaling [77]. It was reported that two paralogous proteins, BSK1 and BSK3, interact directly with BRI1 in the absence of BR, whereas upon the ligand binding to BRI1 this kinase phosphorylates BSK1 on Ser-230, inducing its activation and release from the receptor complex [78]. The activated BSK1 interacts with BRI1-Supressor1 (BSU1) phosphatase [44,79] promoting its interaction with the main negative regulator of BR signaling pathway—Brassinosteroid-Insensitive2 (BIN2), which is described later.

Two homologous proteins—Constitutive Differential Growth1 (CDG1) and CDG-like1 (CDL1), both members of the RLCK-VIIc subfamily of cytoplasmic kinases, have been identified as positive regulators of BR signaling and the substrates of BRI1 kinase domain. Upon ligand binding BRI1 phosphorylates CDG1 on Ser-234 activating its kinase domain. The activated CDG1 phosphorylates BSU1 on Ser-764, located in its C-terminal phosphatase domain, what stimulates its activity and ultimately leads to BSU1-mediated BIN2 dephosphorylation [79,80]. Both CDG1 and CDL1 cytoplasmic kinases are suggested to activate BSU1 phosphatase, which in turn inactivates BIN2 kinase [80,81]. It is suggested that the BSK kinases function mostly as a scaffold during the activation of BSU1 phosphatase, whereas the CDG1 and CDL1 kinases are directly responsible for the stimulation of BSU1 activity through phosphorylation [17]. Therefore, CDG1 provides basal signaling level, whereas BSK1 provides a more effective switch controlled by BR level. It was recently proposed that both BSK1 and CDG1 may activate the cytoplasmic PP2A phosphatase, which exhibits dual activity during the BR signal transduction—repression of BRI1 receptor activity and activation of the key transcription factors mediating BR response - BZR1 and BES1 [82].

Two other polypeptides: Transthyretin-like protein (TTL) and TGFβ-Receptor Interacting Protein1 (TRIP1) which are the substrates of the BRI1 kinase have been identified. TTL is a tetrameric, bifunctional protein with decarboxylase and hydrolase activity [83], which is phosphorylated by BRI1 and functions as a negative regulator of BR signaling [46,84]. The exact role of TTL in regulation of this process is not known [33,85], however it has been recently reported that TTL binds kinase-active BRI1 with higher affinity than kinase-inactive BRI1, indicating that TTL may inhibit BRI1 signaling after its activation [15]. The other cytoplasmic substrate of BRI1–TRIP1 is strongly phosphorylated on many residues by this kinase [86]. TRIP1 similarly to TTL is a dual-function protein. It is an essential subunit of the highly conserved eIF3 translation initiation complex. Therefore, it is suggested that BR-dependent phosphorylation of TRIP1 mediated by BRI1 may affect the eIF3 activity or assembly, and in consequence influence translation, although it has not been unequivocally determined [5]. It is suggested that BR signaling may impact the expression of target genes at multiple stages, like chromatin modification, transcription initiation and elongation and translation [87].

Cytosolic *bri1* Suppressor1 (BSU1) phosphatase plays a crucial role in positive regulation of BR signaling by repressing the activity of BIN2 kinase. BSU1 contains N-terminal Kelch-repeat domain and C-terminal phosphatase domain and shows basal level of BIN2-binding and dephosphorylation. Activated BSU1 interacts with BIN2 kinase and inactivates it through dephosphorylation of Tyr-200, which is crucial residue for BIN2 activity [77,79]. It was shown that BSU1 phosphatase is localized both in the cytoplasm and nucleus, however it was reported that BR response is mediated mainly by the cytoplasmic fraction of this enzyme [88]. On the contrary, BIN2, which is the direct target of BSU1 phosphatase, operates mainly in the nucleus [89]. Several BSU1 homologs have been identified and designated as BSU1-like phosphatases (BSLs), which function redundantly with BSU1 to inactivate BIN2 [77,79].

## 8. BIN2 Kinase Is a Major Negative Regulator of BR Signaling Inactivating Transcription Factors Mediating BR-Dependent Gene Expression

A crucial role in BR signaling is played by the serine-threonine kinase Brassinosteroid-Insensitive2 (BIN2), which is another negative regulator of BR signaling, phosphorylating and thus inhibiting transcription factors regulating expression of target genes. BIN2 protein sequence share similarity with *Drosophila melanogaster* Shaggy kinase and mammalian Glycogen Synthase Kinase3, which often are negative regulators of signaling pathways, regulating different aspects of metabolism [90]. The Arabidopsis *BIN2* belongs to a multi-gene family encoding GSK3-like kinases, regulating broad spectrum of physiological processes, including development of generative organs and responses to salinity stress and wounding [91,92]. BIN2 is encoded by a member of the subfamily of ten related genes—*Arabidopsis Shaggy-like Kinases* (*ASKs*). Several of them, *i.e.*, BIN2 (ASKη), ASKι and ASKζ, which belong to the subgroup II are redundantly involved in the BR signal transduction through phosphorylation of transcription factors regulating BR-dependent gene expression [93–95]. A novel negative regulator of BR signaling, belonging to the subgroup III of the GSK3/Shaggy-like kinase family, has been identified. The new factor—Arabidopsis Shaggy-like Kinase Theta (ASKθ) is a nuclear-localized kinase, which is negatively regulated by BRI1 and phosphorylates the transcription factors BZR1, BES1 and BEH2. It has been reported that ASKθ kinase activity, rather than its protein level is crucial for the BR signaling regulation. The ASKθ kinase functions redundantly with other memebers of the ASK family in mediating BR signaling, however with slightly different binding affinities to the target transcription factors [95].

In the absence of BR BIN2 autophosphorylates on Tyr-200 residue, which is required for its kinase activity [44]. BIN2 kinase activity is suppressed by dephosphorylation of the Tyr-200 residue after perception of BR molecule by the BRI1-BAK1/SERK3 receptor complex and initiation of the signaling cascade [33,96–98]. BIN2 activity is directly inhibited by BSU1 phosphatase, which dephosphorylates the Tyr-200 residue of BIN2 kinase [44]. Another BR-stimulated mechanism of deactivation of the BIN2 kinase has been proposed – the dephosphorylated BIN2 is directed to the proteasomal degradation [40,99].

BIN2 inactivates two closely related transcription factors - Brassinazole-Resistant1 (BZR1) and BRI1-EMS-Supressor1/Brassinazole-Resistant2 (BES1/BZR2) that bind to the promoters of BR-regulated genes [100,101]. These transcription factors belong to plant-specific protein family of six related members, including BES1-Homologues (BEH1-BEH4), which are dephosphorylated in response to BR [102,103]. Apart from these transcription factors, BIN2 phosphorylates CESTA transcription factor belonging to the basic Helix-Loop-Helix (bHLH) family. CESTA positively regulates expression of the BR-biosynthesis *CPD* gene by heterodimerization with the close homologue of CESTA, BRI1 Enhanced Expression1 (BEE1). BIN2-mediated phosphorylation of CESTA is assumed to regulate the nuclear localization of this transcription factor [104]. Based on the results derived from several different approaches it has been suggested that BIN2 operates both in the nucleus and cytoplasm, and the exact mechanism may depend on developmental stage, tissue type and *BIN2* gene expression level [105].

The phosphorylation of the BZR1 and BES1 transcription factors by BIN2 in the nucleus reduces their affinity to DNA, therefore attenuating binding to the promoters of target genes. Additionally, phosphorylated BZR1 and BES1 are also prevented from dimerization with other transcription factors [89]. It was also demonstrated that phosphorylated BZR1 and BES1 are bound by the phosphoprotein-interacting 14-3-3 proteins. Polypeptides belonging to the group 14-3-3 function as another components of the BR signaling with dual role in regulation of this process. Recently, it has been reported that the 14-3-3 proteins may play a positive role in BR signaling by promoting BKI1 dissociation from the plasma membrane, what in consequence results in repressing of the BKI1 inhibitory effect on the BRI1 receptor. Thus, bearing in mind the positive role of the phosphorylated BKI1 in BR signaling, mediated by their binding to 14-3-3 proteins, both negative regulators, BKI1 and 14-3-3 proteins, are upon the BR perception converted to positive regulators of BR signaling by repressing each other [75]. However, the main role of the 14-3-3 proteins in BR signal transduction is based on negative regulation of BZR1 and BES1, mainly by influencing on subcellular localization of these transcription factors. It is suggested that the 14-3-3 proteins may participate in retention of the phosphorylated forms of BZR1 and BES1/BZR2 in the cytoplasm. It was also reported that binding of the phosphorylated forms of BZR1 and BES1 by the 14-3-3 proteins may also result in export of these transcription factors from the nucleus. In consequence, it may lead to the BR-dependent nucleo-cytoplasmic shuttle. The export of these phosphorylated transcription factors into the cytoplasm may be mediated by Exportin1 (CRM1/XPO1). However, it was shown in fractionation studies that phosphorylated form of BZR1 is more abundant in membrane fractions than unphosphorylated form of this protein [88,106–109]. Therefore, the function of the 14-3-3 proteins in BR signaling pathway seems quite complex also because these proteins may have various impact on regulated protein. The 14-3-3 proteins are highly conserved phosphopeptide-binding factors and regulate many important physiological processes through participation in various signaling pathways. Binding of the 14-3-3 proteins usually alters stability, enzymatic activity, affinity to other proteins and subcellular localization of their target proteins [110].

**Figure 2:**
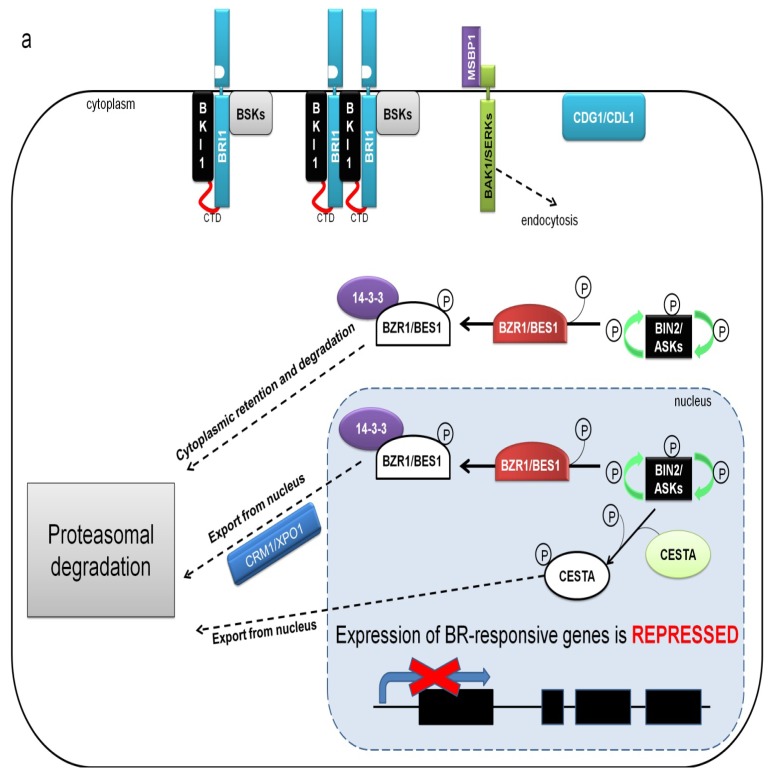

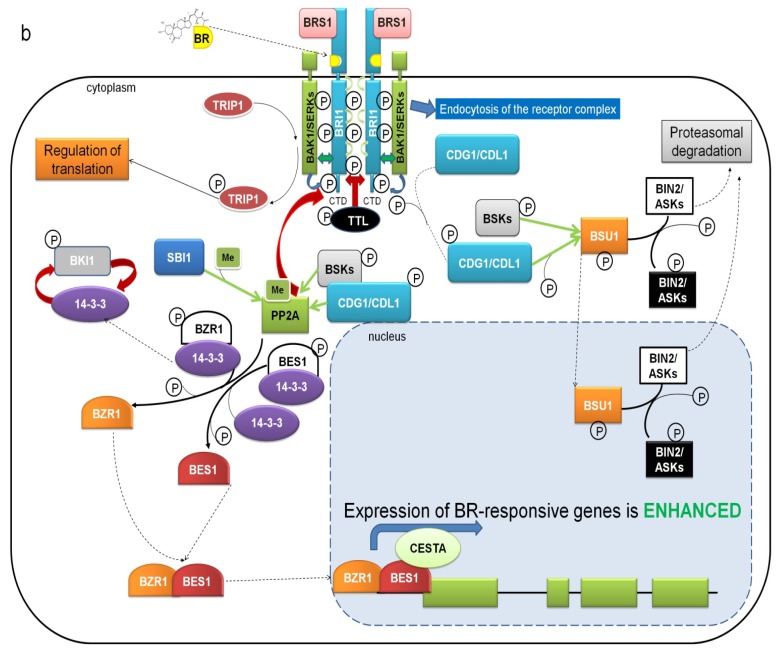
Model of BR perception and signaling pathway. (**a**) In the absence of BR, formation of the receptor complex is inhibited by the C-terminal domain (CTD) of BRI1 kinase and by the BKI1 protein. Positive regulators of BR signaling are inactive, whereas the cytoplasmic BIN2 kinase phosphorylates and inactivates transcription factors, what results in their cytoplasmic retention, export for nucleus and degradation. Expression of the BR-responsive genes is repressed; (**b**) After BR perception by the BRI1 kinase the transmembrane receptor complex is formed and phosphorylation/dephosphorylation cascade is initiated. BIN2 kinase is inhibited, what results in accumulation of active, dephosphorylated forms of transcription factors in the nucleus and stimulation of the expression of BR-responsive genes. The green arrows denote activation, whereas the red arrows denote inhibition; ‘P’ denotes phosphorylation/dephosphorylation, whereas ‘Me’ stands for methylation.

The phosphorylation of BZR1 and BES1 mediated by BIN2 kinase located in the cytoplasm results mainly in cytoplasmic retention of these transcription factors, which is caused by binding the 14-3-3 proteins [77,106,107]. It was shown that phosphorylation on Ser-171 and Thr-175 of BES1 and Ser-171 and Thr-177 of BZR1 are important for the interactions with the 14-3-3 proteins and for the nuclear export. The nuclear export of BZR1 and BES1 is necessary for complete repression of BR signaling [44,109]. Ultimately, phosphorylation of BZR1 and BES1 directs these transcription factors to proteasomal degradation [111]. It has been demonstrated that BIN2 binds BZR1 and BES1 directly though 12-amino acid docking motifs adjacent to their C-terminal ends and these domains are crucial for the interaction, as their deletion leads to constitutive accumulation of the active, dephosphorylated BZR1 and BES1 in the nucleus [92]. An up-to-date and comprehensive model of the BR signal transduction encompassing the newly identified factors is presented in Figure 2.

## 9. BZR1 and BES1 Are Activated by PP2A Phosphatase-Mediated Dephosphorylation

BR perception by the BRI1-BAK1/SERK3 receptor complex causes accumulation of de-phosphorylated and vanishing of phosphorylated forms of BZR1 and BES1/BZR2, which is caused by the inactivation of the BIN2 kinase. Dephosphorylation of BZR1 and BES1/BZR2 transcription factors leads to dissociation of the 14-3-3 proteins and migration of these transcription factors to the nucleus [89,106]. The nuclear localization of the BZR1 and BES1/BZR2 transcription factors is precisely regulated by the level of their phosphorylation, which is determined by the balance in antagonistic activities of BIN2 kinase and recently characterized cytoplasmic PP2A phosphatase. The PP2A phosphatase is composed of the three subunits: scaffolding, regulatory and catalytic one. The regulatory subunit B is responsible for binding to the Pro-Glu-Ser-Thr (PEST) domain of BZR1 and BES1. This domain is of crucial importance both for binding of PP2A phosphatase and for PP2A-mediated dephosphorylation of the residues phosphorylated by the BIN2 kinase. A crucial effect of PP2A on BZR1 and BES1 is a relief of the 14-3-3-mediated cytoplasmic retention of these transcription factors. Therefore, PP2A acts as a positive regulator of BR signaling through dephosphorylation of the BZR1 and BES1 transcription factors, which stimulates their activation and accumulation in the nucleus, and consequently the BR-induced regulation of target gene expression [44,112]. Thus, it may be concluded that PP2A phosphatase is a dual-function regulator of the BR signaling: it regulates positively the signaling through dephosphorylation of the BZR1 and BES1 transcription factors, which leads to activation of these factors and their subsequent nuclear localization, what results in BR-dependent gene expression regulation. On the other hand, the PP2A phosphatase participates in BR signaling feedback regulation by dephosphorylation of the BRI1 receptor.

## 10. The Transcription Factors BZR1 and BES1 Are the Key Regulators of BR-Dependent Gene Expression

The active, dephosphorylated BZR1 and BES1 regulate the expression of hundreds of BR target genes [5,111]. These transcription factors share 88% protein sequence identity and bind DNA through conserved N-terminal domain. Both transcription factors have atypical bHLH domain and function redundantly in BR signaling [101,103]. Both transcription factors, BZR1 and BES1, contain conserved C-terminal fragments, which function as transcription regulation domains [44] and mediate both gene up- and down-regulation, depending on specific target gene promoter and dimerization partner [5]. Both transcription factors, BZR1 and BES1, have similar specificity of target sequence binding, with high affinity for the BR-Response Element (BRRE, CGTG^T^/_C_G) but lower affinity for the E-box element (CANNTG). The E-box elements are present in the promoters of both BR-induced and BR-repressed genes, whereas BRREs are more abundant in the promoters of BR-repressed genes [113]. Both transcription factors share very similar functions, but mediate overlapping yet distinct functions by regulating expression of the overlapping sets of genes. BZR1 regulates expression of more than 80% of the BR-responsive genes and its activating or repressing transcriptional impact is determined by the target gene promoter structure. Moreover, BZR1 homodimer inhibits gene expression through binding to the BRRE elements. It is also speculated that heteromerization of BZR1 with other transcription factors stimulates gene expression by binding to other motifs. BZR1 and BES1 down-regulate expression of several genes involved in BR synthesis as well as the *BRI1* gene based on the feedback loop. Additionally, BES1 down-regulates expression of its two homologs–BEH1 and BEH2 [113]. On the other hand, BZR1 stimulates downstream BR signaling by repressing expression of the *BIN2* gene and enhancing expression of the *BSU1* gene [114]. Negative effect of BZR1 on the expression of *DWARF4* gene, involved in BR biosynthesis, is antagonized by positive effect of the TCP1 transcription factor belonging to the broad family of proteins containing the bHLH domain. BR treatment significantly stimulates the *TCP1* gene expression. However, TCP1 does not bind BZR1 promoter [115,116].

BES1/BZR2 activates transcription of BR-responsive genes together with the group of three BES-Interacting Myc-like proteins (BIM1-3), which interact with BES1 by binding to its HLH dimerization domain [44,56,101,114]. In response to BRs BZR1 and BES1 bind their own promoter sequences and induce their own expression through a positive feedback loop [113]. In addition to BZR1 and BES1, several other DNA binding proteins are implicated in BR signaling. Specificity and strength of promoter binding and the extent of transcription activation mediated by BES1 is regulated by their association with other transcription regulators, belonging to the various subfamilies: bHLH, MYB, IWS and Jumonji N/C domain [87,103,117,118]. This group includes several auxin-regulated proteins, Myb transcription factors, GRAS-family proteins, proteins modulating chromatin structure and the group of three bHLH proteins – BRI1 Enhanced Expression (BEE1-3) [119]. It was shown that BES1 recruits histone demethylases and transcription elongation factors to regulate gene expression [44]. Transcriptional activity of BES1 is enhanced by two factors—EARLY FLOWERING6 (ELF6) and RELATIVE OF EARLY FLOWERING 6 (REF6). Both these factors contain highly conserved Jumonji N/C domain characteristic for histone H3 demethylases. BES1 interacts with these factors to regulate BR responses and other physiological processes, such as flowering [120,121]. Full transcriptional activity of BES1 requires association with the Interacting-With-Spt6 1 (IWS1) factor, known to regulate RNA polymerase II postrecruitment, transcriptional elongation, RNA export and histone modifications [87,122]. BES1 recruits IWS1 to transcribed regions of the BES1 target genes to enhance transcription elongation [120]. Apart from dimerization with other transcription factors, BZR1 and BES1 regulate the expression of numerous transcription factors by binding directly to their promoter sequences, what results in secondary amplification of BR-regulated transcriptional response [5,114]. This mechanism is illustrated by the representative of the MYB factors—the *MYB30* gene, which is a direct target of BES1 transcription factor and itself regulates a subset of BR-responsive genes [118]. MYB30 interacts with its regulator, BES1, and these associated transcription factors regulate the expression of BR-target genes, which is an example of the signal amplification [120].

As far as the extent of BR-dependent regulation of gene expression is concerned, it was reported that expression of about 1,200 genes is influenced by BRs. Out of this number about 950 genes proved to be direct targets of BZR1 transcription factor, 450 of them were up-, whereas another 462 genes were down-regulated [114]. About 250 genes exhibiting BR-dependent expression were shown to be direct target of BES1. Out of this group, about 160 genes were up-regulated, while the expression of the rest of them was attenuated [113]. The groups of BZR1 and BES1 target genes showed an overlap of 120 genes, indicating that common regulation of the gene expression by these transcription factors takes place [5].

## 11. Newly Identified Transcription Factors Regulating the BR-Dependent Gene Expression

Recently, a new key factor regulating BR-dependent gene expression has been identified. It has been reported that BES1 interacts with the Myeloblastosis family transcription factor-like 2 (MYBL2), which is a transcription repressor, inhibiting expression of BR-repressed genes. The loss-of-function mutation of the *MYBL2* gene results in a typical phenotype of mutant plant with defects in BR metabolism, what suggests that suppression of BR-repressed gene expression is crucial for optimal BR response. Interestingly, MYBL2 is a substrate of BIN2 kinase. However, unlike BIN2 phosphorylation of the BZR1 and BES1 transcription factors, which renders these factors inactive and directs them to proteasomal degradation, it was shown that BIN2 phosphorylation stabilizes MYBL2 [123]. Hence, phosphorylation mediated by BIN2 kinase may have both stabilizing and destabilizing effect, depending on the substrate of phosphorylation.

The novel transcription factor, Related to ABI3/VP1 (RAV1), has been identified in Arabidopsis and rice, which stimulates expression of both the BR-receptor gene *BRI1* and several BR-biosynthesis genes, however the activation of expression of the BR-synthesis genes mediated by this transcription factor may be suppressed by BZR1. RAV1 transcription factor contains B3 DNA-binding domain showing different affinity for E-box elements in the promoters of the regulated genes. Surprisingly, RAV1 is not subject to BR feedback regulation, however is involved in maintaining the expression of the regulated genes implicated in BR signaling and synthesis at the basal level. RAV1 expression is suggested to be negatively regulated by auxins through binding of the Auxin Response Factor (ARF) to the RAV1 promoter. This mechanism together with the negative feedback regulation maintains BR homeostasis [124,125].

A new group of atypical bHLH transcription factors, comprising six proteins PACLOBUTRAZOL RESISTANT (PRE1–6) is implicated in positive regulation of BR signaling [119,126]. Expression of one of the members of the PRE family—PRE1 is enhanced by BZR1 by its direct binding to the promoter of the *PRE1* gene. Activity of PRE1 (and maybe the other PRE paralogs) is based on mechanism, in which dimerization of the transcription factor with classical bHLH protein promotes binding to the E-box motif (CANNTG) in promoter of a target gene, whereas association with atypical, non-DNA binding bHLH factors inhibits DNA binding of both associated transcription factors [127]. It is suggested that PRE1 interacts with the classical bHLH protein, ILI1 Binding bHLH Protein1 (IBH1), which is thought to be a negative regulator of BR-dependent gene expression. PRE1 attenuates the negative effect of IBH1 by dimerization and consequently inhibition of DNA binding by IBH1. PRE1 and IBH1 exhibit different expression patterns—PRE1 is highly expressed in tissues at early developmental stages, whereas IBH1 shows more pronounced expression in mature organs. It was also shown that IBH1 is a direct target of the BZR1 transcription factor, however contrary to the *PRE1* gene expression regulation, BZR1 exerts negative effect on the expression level of IBH1. Therefore, the antagonistic activity of PRE1 and IBH1 may constitute a molecular mechanism that stimulates growth rate in tissues at early developmental stages, which are known to be more BR-sensitive, whereas in mature organs growth is inhibited [120,126].

**Figure 3:**
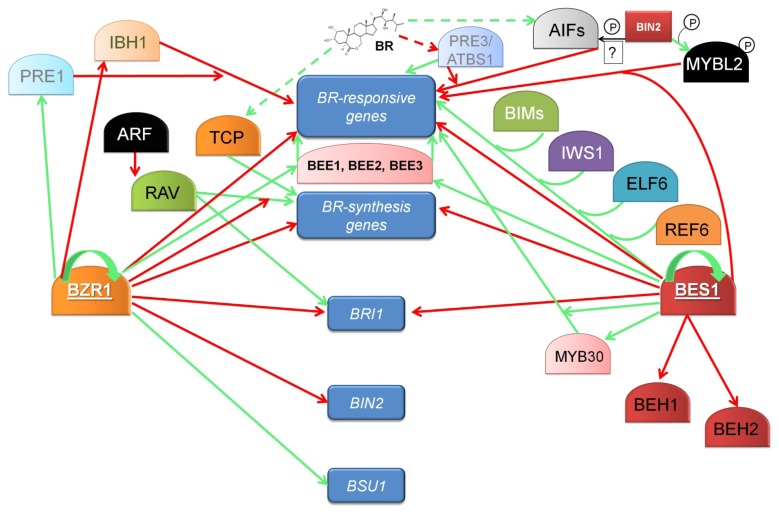
Model of BR-dependent regulation of target gene expression, mediated by the network of transcription factors. The names of the BR-regulated genes and gene groups are italicized and shown in the blue rectangles. Green arrows show positive regulation, whereas red arrows denote repression. Black arrow indicates that function of BIN2-mediated phosphorylation of the AIFs transcription factors is currently unknown; ‘P’ denotes phosphorylation events.

The same mechanism based on the dimerization of transcription factors, which influences the expression of target genes has been proposed for another representative of the PRE family—PRE3, also named Activation-Tagged BRI1-Suppressor1 (ATBS1), which is a nuclear-localized positive regulator of BR-dependent gene expression. It has been shown that ATBS1 interacts with four paralogous, nuclear-localized atypical bHLH transcription factors ATBS1-Interacting Factor (AIFs), which are known to be negative regulators of BR-dependent gene expression, incapable of binding DNA. It is suggested that AIFs function through interactions with two BEE proteins, but not with the BZR1 or BES1 transcription factors. Positive role of ATBS1 in the regulation of BR signaling is mediated by sequestering the negative regulators—AIFs. This model grew even more complicated by the fact of AIF1 phosphorylation by BIN2 kinase, but the exact effect of this phosphorylation on AIF1 activity is not fully understood [119,120]. Expression level of the *ATBS1* and *AIF* genes is BR-dependent, however these genes show contrary expression profiles—*ATBS1* expression is down-regulated, while expression of *AIF* is induced by BR [9]. Moreover, expression of *ATBS1* is directly regulated by auxin-dependent transcription factor MONOPTEROS. Other PRE factors are implicated in gibberellin and light signaling pathways as well as photomorphogenesis [128–130] indicating that members of the PRE family may constitute of point of interactions of several signaling pathways, namely BR, auxin, gibberellin and light signal transduction relays. All this clearly indicates that transcriptional regulation of BR-responsive genes is much more complicated than what could be expected from linear signaling pathway involving only two main transcription factors [119,120]. It is now clear, that that the mechanism of transcriptional regulation of genes expression influenced by BR has a structure of network, which recently became very complicated and not fully understood. The network of transcriptional regulation of BR-dependent gene expression is presented in Figure 3.

## 12. Several Mediators of BR Signaling Are Involved in Other Signalosomes and Function as the Points of Cross-Talk between Pathways Regulating Various Physiological Processes and Stress Responses

Pathogen attacks pose a major factor limiting seed production [14]. During evolution, plants have evolved a complex system of innate immunity leading to pathogen resistance. Plants perceive pathogen/microbe-associated molecular patterns (PAMPs/MAMPs) through deployment of the Pathogen-Recognition Receptors (PRRs), what results in initiation of PAMP-Triggered Immunity (PTI) [131,132]. The PAMP perception causes a series of downstream events including ion fluxes, increase in concentration of the reactive oxygen species (ROS) resulting in oxidative burst, stimulation of Mitogen-Activated Protein Kinase (MAPK) pathway and induction of calcium-dependent protein kinases. All these reactions lead to induction of defence gene expression [133,134]. Eventually, these events lead to the PAMP-triggered immunity and resistance to a range of pathogens [135]. Additionally, certain plant species detect pathogen-derived effectors by the Resistance (R) proteins and initiate Effector-Triggered Immunity (ETI) resulting in strong defence reactions, constituting a form of programmed cell death [68]. In Arabidopsis two well-characterized and phylogenetically related transmembrane PRRs: Flagellin-Sensing 2 (FLS2) and EF-Tu (EFR) receptors bind bacterial peptides derived from flagellin and elongation factor Tu, respectively. These receptors belong to the LRR-RLK family and initiate defence responses involving the induction of the MAPK pathway [136–138]. Both FLS2 and EFR are crucial for plant antibacterial immunity [139].

Recent studies clearly indicated, that some of the components of BR signaling pathway act as multifunctional proteins involved in other signaling networks regulating diverse physiological processes, like photomorphogenesis, cell death control, stomatal development, flowering, plant immunity to pathogens and metabolic responses to stress conditions. Regulation of some of these processes is mediated through a crosstalk between BR signaling pathway and signaling cascades of other hormones, including auxin, abscisic acid, ethylene and salicylic acid [14]. One of the best examples is the participation of the SERK proteins and BAK1/SERK3 in particular, whose function in BR signaling has been described earlier in this review, in regulation of such diverse physiological processes as cell-death control and numerous pathogen-associated responses to various elicitors, *i.e.*, flagellin, the elongation factor EF-Tu, the bacterial-cold shock protein and the oomycete elicitor INF1. This molecular responses act as the first line of defence against pathogens [62,63,71,73,140].

BAK1/SERK3 is a convergence point that connects growth promoting BR responses with PAMP signalosome [76,141]. Perception of the flagellin peptide by FLS2 promotes metabolic processes leading to the suppression of pathogen proliferation [62,140,142]. BAK1 was found to associate with several receptors perceiving pathogen/microbe-associated molecular patterns, including FLS2 and EFR, shortly after binding the pathogen elicitors. Complex formation between FLS2 and BAK1 is solely triggered by the interaction of FLS2 with its ligand [66]. Similar to BR signaling, the flagellin perception by FLS2 leads to transphosphorylation with BAK1/SERK3 and mutual activation. The activated FLS2 phosphorylates BIK1 (described below) and initiates signaling cascade. This sequence of events is crucial for full activation of the pathogen-induced defence responses, including stimulation of MAPK signalosome and production of the reactive oxygen species. Therefore, BAK1/SERK3 is a co-receptor recruited upon ligand perception by several LRR-RLKs mediating various signaling pathways [143–145]. The effect of *BAK1* loss-of-function on the EFR-mediated response is less pronounced than on the FLS2-regulated signaling pathway [68,140,146]. It is suggested that such a versatile activity of this kinase as the component of several different receptor complexes results from differential phosphorylation patterns induced in BAK1 by perception of different ligands. It was reported that Thr-450 of BAK1 is phosphorylated by BRI1 after ligand binding and is crucial for BR signaling. However, when substituted by another residue does not affect the various BAK-dependent signaling pathways to the same extent [141]. It was reported that both FLS2 and EFR recruit other members of the SERK family in the ligand-induced manner. The SERK proteins BAK1/SERK3 and BKK1/SERK4 cooperatively and redundantly regulate multiple PRR-mediated signalosomes. Both of these RLKs play a crucial role in disease resistance against hemibiotrophic bacteria and obligate biotrophic oomycete. The two other SERK proteins—SERK1 and SERK2 were also found to interact with the FLS2 and EFR receptors. Additionally, transcription of the *SERK* genes is up-regulated in response to PAMP and pathogen treatment [65], what supports the role of these factors in the innate immunity. It was reported that BAK1/SERK3 is a preferable interactor of FLS2, while EFR is less selective for a particular SERK proteins and interacts with the SERK proteins with equal affinity. However, the composition and stoichiometry of these receptor complexes have not been determined yet [146]. It has been also shown that BAK1/SERK3 and BKK1/SERK4 are also recruited by the PEP Receptors 1 and 2 (PEPR1 and PEPR2), which bind PAMP- and wound-induced endogenous peptides, and act as amplifiers of the PTI response [147,148]. Thus, these SERK proteins are required to regulate various stress responses mediated by the EFR, FLS2 and PEPR receptors and downstream signaling events [146]. Recently the interplay between the plant immune system and BR metabolism has been extensively studied. The results of this approach demonstrated that BR metabolism is a rate-limiting modulator of BAK1-mediated PAMP responses. BR-independent function of BAK1 in plant immune response is impacted by its BR-dependent role in plant growth and development [145]. The signaling pathways of brassinosteroids and PTI response are linked in unidirectional antagonistic manner [149]. BR-stimulated BRI1-BAK1 heterodimerization represses FLS2-mediated signaling by decreasing the amount of available BAK1. Additionally, downstream cytoplasmic components of BR signalosome, such as BIN2, BZR1 and BES1 may also suppress plant immunity signaling downstream of BIK1, however the exact mechanism has not yet been fully elucidated [76,150]. It was recently reported that BAK1/SERK3 plays also a crucial role in mediating resistance of *Nicotiana attenuata* plants against its herbivore *Manduca sexta*. This mechanism is dependent on modulation of the herbivory-induced jasmonic acid accumulation and regulation of activity of defensive secondary metabolites [151].

BAK1/SERK3 is also implicated in cell-death control, and *bak1* knock-out mutants have a spreading lesion phenotype upon pathogen infection and premature senescence. This phonotype is further enhanced in double-mutant combinations with its closest paralogue BKK1/SERK4, also mediating BR perception. Interestingly, cell-death phenotype of the double mutant *bak1/bkk1* occurs even in a sterile growth conditions. It was also reported that the phenotype is enhanced by light, therefore it is suggested that the double mutant is deprived of the capability to detoxify or restrict the production of phototoxins induced by light. The uncontrolled accumulation of the phototoxins is likely a cause of the observable spontaneous cell death phenotype [71,73,152,153]. BAK1/SERK3 interacts with BAK1-Interacting Receptor-like kinase (BIR1) to negatively regulate cell death and defence responses. Interestingly, unlike BRI1 or FLS2 receptors BIR1 contains only five LRRs. Taken together, BAK1 functions as a positive regulator of PAMP-triggered immune response, however this kinase is a negative regulator of effector-triggered immunity mediated by the host Resistance (R) protein, which is aimed at inhibition of pathogen growth and its spread through localized cell death [154].

Identification and characterization of the new BAK1 allele, *bak1-5*, demonstrated that the role of BAK1 in various signaling pathways is mechanically separated. The missense mutation in BAK1 kinase domain represses innate immune response, whereas steroid signaling and cell-death control remain unaffected [155]. Another BAK1 allele, *elongated-D* (*elg-D*), exhibits enhanced BR response through elevated level of BR signaling. This mutant harbours change of the highly conserved Asp-122 in the third LRR of BAK1 co-receptor, which renders the protein constitutively active in the stimulation of BR response. Apart from the increase in BR signaling, *elg-D* allele causes increased efficiency of infection with *Pseudomonas syringae*, whereas the allele showed no effect on EFR-mediated signaling. Therefore, it is concluded that this allele of *BAK1* leads to the promotion of plant growth, mediated by the elevated level of BR signaling, at the expense of efficiency of the responses to pathogen invasion and salinity stress, which are impaired. It substantiates the general observation that increased growth often impairs the plant resistance to stress conditions [48,156]. It is suggested that *elg-D* allele stimulates BR signaling by causing the encoded mutated BAK1 co-receptor to associate preferentially with BRI1 over FLS2, most likely by enhancing the ‘double-lock’ interaction. This affinity difference underlies the opposite gain- and loss-of-function phenotypes in BR and flagellin signaling, respectively [47]. Therefore, BAK1 as the multifaceted co-receptor mediating various signaling pathways, regulating the cell growth and immune responses, is a hub decision point determining whether to utilize the resources to initiate pathogen-defence response or to stimulate plant growth to gain access to new resources, *i.e.*, light, water or nutrients [157].

It has been reported that two unrelated effectors from *Pseudomonas syringae*, AvrPto and AvrPtoB, target early steps in various PAMP pathways, upstream the MAPK cascade important for Pathogen-Triggered Immunity (PTI) [158]. Both effectors bind with high affinity to BAK1 and prevent its ligand-dependent association with FLS2 [159]. Interestingly, overexpression of both factors results in BR-related plant dwarfism, caused by repression of BAK1 interaction with BRI1. This would indicate that BR signaling is reduced upon infection, and suggests a model in which the bacterial effectors attenuate BR signaling to improve efficiency of infection [141].

It has been recently reported that the SERK proteins are not the only members of the BR receptor complex that serve as the cross-talk points with other signaling pathways. It has been shown that calmodulin (CaM) interacts with the cytoplasmic kinase domain of BRI1 receptor in Ca^2+^-dependent manner [160]. It is now well established that calcium ions are ubiquitous second messengers, which mediate various metabolic responses. In plants several biotic and abiotic stimuli including phytohormones, oxidative stress and microbial elicitors induce alterations in cellular Ca^2+^ concentration [161,162]. Calmodulin proteins are sensors of the changes in concentration of calcium ions, which mediate cellular responses. Binding of Ca^2+^ to calmodulin induces its conformational changes, which enable interacting with target proteins leading to alteration of their activities [163,164]. It was shown that Ca^2+^-dependent CaM binding to the kinase domain of BRI1 inhibits both auto- and transphosphorylation activity of the receptor kinase. The interaction of calmodulin and BRI1 is suggested to constitute a link between BR and Ca^2+^ signalosomes [160].

It has been recently reported that Brassinosteroid Signaling Kinase5 (BSK5) belonging to the RLCK family, the same to which two paralogous factors, BSK1 and BSK3 mediating BR signaling belong, is up-regulated by BR and ABA. The abiotic stresses, including salinity and drought, also enhance BSK5 expression to various extents. BSK5 kinase is required for salt stress and ABA-mediated drought stress tolerance. BSK5 stimulates salt stress response and suppresses salinity-induced ABA biosynthesis. This kinase regulates process of ABA-dependent stomatal closure under drought stress and negatively influences drought stress response through modulating gene expression [165].

Similarly to the members of the SERK family, several members of the RLCK-VII subfamily, the one to which CDG1 belongs, including plasma membrane-localized protein PBS1, PBS-like proteins (PBLs) and Botrytis-Induced Kinase1 (BIK1) function downstream of multiple immune receptor kinases perceiving pathogen-associated molecular patterns. PBL1 and BIK1 interact directly with the unstimulated flagelling receptor FLS2 and BAK1. Upon binding of flagellin by the FLS2 receptor PBL1 and BIK1 become phosphorylated, and as a result they transphosphorylate FLS2 and BAK1 and dissociate from the receptor complex [143,144]. Thus, BIK1 is suggested to be the component of the BAK1-mediated signalosome that regulates resistance against pathogens [68]. Therefore, it is concluded that BRI1 and FLS2 receptors share not only the same co-receptor kinase—BAK1 [67], but also the members of the same RLCK family, which mediate downstream signaling relays.

Recently it has been reported that BRs are also implicated in the regulation of stomatal development. The regulatory function is based on activation of the MAPK kinase kinase YODA, which is a negative regulator of stomatal development. In the absence of BRs, BIN2 kinase (the key negative regulator of BR signaling) inhibits YODA activity through phosphorylation of its auto-regulatory domain, leading to suppression of the MAPK signaling [166,167]. It brings about derepression of SPEECHLESS (SPCH), which initiates stomatal development. BRs inhibit stomatal development through repression of BIN2 activity what results in activation of the MAPK relay [76,167]. It is suggested that BIN2 mediates phosphorylation-dependent regulation of both key factors of the stomatal development—YDA and SPCH [168].

BIN2 kinase activity is not merely restricted to BR signaling pathway, it also phosphorylates Auxin Response Factor 2 (ARF2), which is a negative regulator of auxin response. The BIN2-mediated phosphorylation inhibits DNA-binding activity of ARF2, hence inducing downstream auxin responses [169]. The direct interaction between BIN2 and ARF2 represents an example of the synergistic effects of BRs and auxins. The synergistic interaction between these two hormones is also illustrated by the fact, that auxin represses negative effect of the BZR1 transcription factor on expression of the BR biosynthesis gene *DWF4* [76,169]. BIN2 seems to function as a negative regulator of several proteins involved in distinct signalosomes, including BR-, auxin- and stomatal development-related relays [170], thus BIN2 may pose a point of inter-connections of several signaling pathways regulating plant growth and development.

Brassinosteroids enhance flowering by stimulating the expression level of the *LD* and *FCA* autonomous pathways genes, which suppress the *FLC* gene expression. However, it is suggested that BR promote flowering also independently of the *LD* and *FCA* genes and this pathway is also aimed at BR-induced suppression of the *FLC* expression [121]. BES1 transcription factor, being one of the key players of the BR-dependent regulation of gene expression, regulates expression of *ELF6* and *REF6*, which are involved in the flowering time control. *ELF6* functions in the photoperiodic flowering pathway, whereas *REF6* acts as a repressor of the *FLC* gene expression [117]. It was also reported that BES1 represses expression of two related transcription factors, GLK1 and GLK2, which are involved in chloroplast development. It is suggested, that this activity underlies BR-mediated inhibition of chloroplast development in the dark [113]. Apart from the involvement of BES1 in the signal transduction regulating flowering time and photomorphogenesis, this transcription factor is also implicated in the pathogen-triggered response pathway. BES1 regulates expression level of the MYB30 transcription factor, which mediates hypersensitive response to pathogen attack [171], what indicates that BES1-regulated expression of MYB30 is yet another point of cross-talk between BR signaling and pathogen-triggered molecular response in which BR stimulates pathogen resistance [120].

Another key regulator of BR-dependent gene expression, BZR1 mediates signaling pathways cross-talk between BR and auxin by direct regulation of auxin-responsive genes expression as well as modulation of expression of multiple genes involved in auxin signaling and metabolism. It was also reported that both transcription factors BES1 and BZR1 bind to the promoters of numerous genes involved in the signaling and synthesis pathways of gibberellin (GA), abscisic acid, ethylene, cytokinin and jasmonate, suggesting that BR signaling impacts metabolism of several other plant hormones [113,114]. It was recently reported that BRs and GA act interdependently through a direct interaction between two transcription factors—the BR-activated BZR1 and GA-inactivated DELLA. Stimulatory effect of GA on cell elongation requires BR signaling, on the other hand BR-activated BZR1 supresses GA-deficient dwarf phenotype. The DELLA transcription factors directly interact with BZR1 and inhibit BZR1-DNA binding. Genome-wide analysis of BZR1-dependent GA-regulated transcriptome indicated that it includes genes involved in cell wall synthesis, as well as light-regulated genes and those related with photosynthesis and chloroplast function. GA-induced promotion of hypocotyl elongation is mediated by BZR1 and the phytochrome-interacting factors (PIFs), as well as their common downstream targets encoding the PRE-family factors. It indicates that GA releases DELLA-mediated inhibition of BZR1 and that the DELLA-BZR1-PIF interaction constitutes a core transcriptional network, which modulates coordinated growth regulation by GA, BR and light [172]. The direct interaction between the dark- and heat-induced PIF4 transcription factor, and the BR-activated BZR1 integrates the hormonal and environmental signals. BZR1 and PIF4 interact with each other and bind to nearly 2,000 promoters of common target genes. These two transcription factors synergistically regulate many of the target genes. BZR1 and PIFs are interdependent in promoting cell elongation in response to BR, darkness and heat stimuli [173].

BZR1 was reported to repress expression of the GATA2 transcription factor, which is the main component of transcriptional regulation of many genes involved in de-etiolation. GATA is a key transcriptional regulator that mediates cross-talk between BR- and light signaling pathways, being positive regulator of photomorphogenesis. This BR-dependent transcriptional repression of the *GATA2* gene is mediated by direct binding of BZR1 to the *GATA2* promoter, and this mechanisms is crucial to prevent photomorphogenesis in the dark. BR signaling efficiency does not fluctuate significantly under light and dark conditions, however BZR1 binding to *GATA2* promoter is reduced in the light, what results in attenuation of the repressive impact of BR on GATA2 expression. Moreover, light stimulates accumulation of GATA2 protein, and on the other hand, inhibits the *GATA2* gene transcription in a feedback manner. GATA2 inhibits its own transcription in a feedback manner, and this process serves as the mechanism of desensitization during transition from dark to light. Taken together, this BZR1-mediated repression of the *GATA2* gene expression is essential for maintaining complete skotomorphogenesis in the dark [174,175]. It has also been shown that BZR1 regulates expression of more than 1,100 target genes, whose expression is also modulated by the basic leucine zipper transcription factor HY5, which is known to be a positive regulator of photomorphogenesis. However, this set of target genes is regulated by these transcription factors in opposite manner, what supports the antagonistic interaction between BR and light signals [114]. Recently a novel B-box transcription factor, BZR1 Suppressor1 (BZS1), which functions downstream of both BR and light signaling pathways has been identified. Expression of this transcription factor is negatively regulated by BR and is mediated by BZR1. Repression of BZS1 transcription by BZR1 contributes to negative effect of BRs on photomorphogenesis. Additionally, transcription level of *BZS1* was decreased in the light, but induced in the dark. On the other hand, light promotes BZS1 protein accumulation. BZS1 represses BR responses, however positively regulates light signaling pathway. It is suggested that BZS1 interacts with HY5 transcription factor to control expression of a subset of light-responsive genes [176].

It is now well known that in response to wide variety of abiotic stresses, *i.e.*, oxidative stress, drought, salinity, heavy metal stress and thermal stress BRs promote expression and activity of many enzymes, including superoxide dismutases, catalases, ascorbate peroxidises, glutathione reductases and heat-shock proteins, as well as enhance concentration of metabolites, *i.e.*, ascorbic acid, carotenoids, glutathione and phytochelatins. Moreover, in response to biotic stresses, mainly evoked by bacterial, fungal and viral infections BRs stimulate synthesis of abscisic acid, ethylene and salicylic acid [177]. BRs regulate several biological processes and stress responses through interactions with other hormones. Regulation of these processes is mediated by common control of the target gene expression [178–180]. BRs promote tolerance to the variety of abiotic stresses, including heat, cold, drought and salinity though the enhancement of expression of stress-related genes including the Heat Shock Proteins (HSPs). BR stimulates stress tolerance in concert with other phytohormones. Vast majority of genes regulated by salicylic acid, jasmonic acid, ethylene and ABA is also up-regulated by BR. This indicates that the various plant hormones regulate overlapping sets of target genes, what results in inter-hormonal interactions in mediating plant stress responses. BRs positively regulate defence gene expression and thermotolerance by enhancing activity of salicylic acid signaling components. Moreover, BRs stimulate salt tolerance directly or through induction of ethylene signaling [181]. It has been recently reported that BR-stimulated salt tolerance is mediated by the Ubiquitin-Conjugating Enzyme32 (UBC32), which regulates cellular accumulation of the BRI1 receptor [182]. BR together with ABA promote stomatal closure, which is mediated by nitric oxide, functioning as a mediator of ABA-induced stomatal closure. BR signaling promotes nitric oxide production, which in turn stimulates ABA synthesis, what results in water stress tolerance. Moreover, BR signaling regulates expression of genes, which are involved in the induction of antioxidant systems, which serve as protectors against deleterious effects of reactive oxygen species. BR perception positively regulates the NADPH oxidase, which regulates the rate of H_2_O_2_ production, what impacts abiotic and biotic stress tolerance, however the exact mechanism is not fully elucidated [76].

BRs belong to a vast group of factors influencing ethylene biosynthesis through regulation of activities of key enzymes involved in this process [183]. Recent report suggests that interaction between BRs and ethylene leads to regulation of the plant defence responses. The fungal Ethylene-inducing xylanase (Eix) proteins belong to very effective elicitors triggering plant defence responses. In *Solanum lycopersicum* Eix protein is perceived by two receptors LeEix1 and LeEix2 belonging to the RLK family. However, LeEix2 is the only receptor initiating defence signaling, whereas LeEix1 heterodimerizes with LeEix2 and attenuates the signaling. Moreover, the inhibitory function of LeEix1 depends on association of this receptor with the BAK1/SERK3 kinase. This indicates that Eix-induced defence response mediated by LeEix2 is attenuated by the interaction of BAK1 with the decoy receptor LeEix1. The kinase activity of BAK1 is required for the attenuation of Eix-induced signaling [64].

## 13. Conclusions

Involvement of BR signaling components in such a wide variety of metabolic pathways regulating diverse physiological processes, including plant responses to biotic and abiotic stresses, indicates that the members of BR signalosome may also serve in the future as target for genetic engineering approaches aimed at improved responses to biotic and abiotic stress factors. In fact, overexpression of the BAK1 homologue (OsSERK1) in rice led to increase in the resistance of transgenic rice plants to blast fungus [184]. Therefore, extensive studies aimed at elucidation of the molecular mechanisms of BR signaling and its interconnections with other signalosomes are still required. The results of this investigations may be of crucial importance for better understanding of plant physiology and stress responses, and consequently for future application in agriculture.
